# Growth of MCF-7 breast cancer cells and efficacy of anti-angiogenic agents in a hydroxyethyl chitosan/glycidyl methacrylate hydrogel

**DOI:** 10.1186/s12935-017-0424-8

**Published:** 2017-05-16

**Authors:** Hejing Wang, Junmin Qian, Yaping Zhang, Weijun Xu, Juxiang Xiao, Aili Suo

**Affiliations:** 1 Department of Oncology, The First Affiliated Hospital of Xi’an Jiaotong University, No. 277, Yanta West Road, Xi’an, Shanxi People’s Republic of China; 2 State Key Laboratory for Mechanical Behaviours of Materials, Xi’an Jiaotong University, Xi’an, 710049 China

**Keywords:** Breast cancer, MCF-7 cells, 3-Dimensional cell culture, Hydrogel

## Abstract

**Background:**

Breast cancer negatively affects women’s health worldwide. The tumour microenvironment plays a critical role in tumour initiation, proliferation, and metastasis. Cancer cells are traditionally grown in two-dimensional (2D) cultures as monolayers on a flat solid surface lacking cell–cell and cell–matrix interactions. These experimental conditions deviate from the clinical situation. Improved experimental systems that can mimic the in vivo situation are required to discover new therapies, particularly for anti-angiogenic agents that mainly target intercellular factors and play an essential role in treating some cancers.

**Methods:**

Chitosan can be modified to construct three-dimensional (3D) tumour models. Here, we report an in vitro 3D tumour model using a hydroxyethyl chitosan/glycidyl methacrylate (HECS–GMA) hydrogel produced by a series of chitosan modifications. Parameters relating to cell morphology, viability, proliferation, and migration were analysed using breast cancer MCF-7 cells. In a xenograft model, secretion of angiogenesis-related growth factors and the anti-angiogenic efficacy of Endostar and Bevacizumab in cells grown in HECS–GMA hydrogels were assessed by immunohistochemistry.

**Results:**

Hydroxyethyl chitosan/glycidyl methacrylate hydrogels had a highly porous microstructure, mechanical properties, swelling ratio, and morphology consistent with a 3D tumour model. Compared with a 2D monolayer culture, breast cancer MCF-7 cells residing in the HECS–GMA hydrogels grew as tumour-like clusters in a 3D formation. In a xenograft model, MCF-7 cells cultured in the HECS–GMA hydrogels had increased secretion of angiogenesis-related growth factors. Recombinant human endostatin (Endostar), but not Bevacizumab (Avastin), was an effective anti-angiogenic agent in HECS–GMA hydrogels.

**Conclusions:**

The HECS–GMA hydrogel provided a 3D tumour model that mimicked the in vivo cancer microenvironment and supported the growth of MCF7 cells better than traditional tissue culture plates. The HECS–GMA hydrogel may offer an improved platform to minimize the gap between traditional tissue culture plates and clinical applicability. In addition, the anti-angiogenic efficacy of drugs such as Endostar and Bevacizumab can be more comprehensively studied and assessed in HECS–GMA hydrogels.

## Background

In developed countries, cancer is the leading cause of death, whereas in developing countries, it is second only to heart disease [1]. Breast cancer is the second most common cancer overall (1.7 million cases, 11.9%) but ranks fifth as the cause of death (522,000, 6.4%) [2]. Substantial research expenditure is applied to develop new anticancer drugs, but most fail in animal models or clinical trials despite showing promise in preclinical testing [3–5].

The tumour microenvironment plays a critical role in tumour initiation, proliferation, and metastasis, and contains potential therapeutic targets [6–9]. Anticancer therapy is undergoing a conversion from a cancer cell-centric to a stroma-centric strategy, mainly because stromal components are more genomically stable [10]. Angiogenesis, induced by the tumour microenvironment, plays an important role in promoting tumour growth and metastasis as well as providing the tumour with sufficient oxygen and nutrients for growth [11].

Researchers have long relied on two-dimensional (2D) in vitro cell culture systems to study cancer cells. In 2D culture systems, cells are grown as monolayers on a flat solid surface lacking the cell–cell and cell–matrix interactions that are present in native tumours. Additionally, 2D cultured cells are exposed to much higher oxygen concentrations than in vivo, and are stretched and undergo cytoskeletal rearrangements acquiring artificial polarity, which in turn causes aberrant gene and protein expression [12]. In contrast, three-dimensional (3D) culture systems offer the unique opportunity to culture cancer cells in a structure that more closely mimics the native environment of tumours [13–15]. 3D culture models also have been used to assess the diffusion, distribution, and efficacy of drugs [16].

In the last few decades, hydrogel scaffolds, which are cross-linked networks that possess high water contents, have attracted increasing attention in an attempt to mimic in vivo conditions [17]. However, synthetic polymers such as polylactide and polyglycolide have large fibre diameters and pore sizes that yield poor scaffold structures with mechanical properties that do not accurately mimic the full complexity of the natural environment of cell growth [18].

Chitosan has been used for the construction of 3D culture models due to its biocompatible, biodegradable, non-immunogenic, and non-inflammatory characteristics [19, 20]. In addition, it has antitumour properties, haemostatic function, and antibacterial activity [21, 22]. However, chitosan is only soluble in dilute acidic solutions, which limits its applications. There has been growing interest in the chemical modification of chitosan to improve its solubility and broaden its applications [23, 24]. However, the selection of appropriate 3D scaffolds and characterization of the physical properties of the scaffolds (such as fibre length, porosity, and stiffness associated with cellular responses) and gene expression profile remain a challenge for the scaffold engineering field and for mimicking the tumour tissue environment [25]. In our research, we describe, for the first time, a method to prepare a novel chitosan derivative: a hydroxyethyl chitosan–glycidyl methacrylate (HECS–GMA) hydrogel.

## Methods

### Materials

Glycidyl methacrylate (GMA), fluorescein isothiocyanate labelled phalloidin (FITC-phalloidin), Dulbecco’s phosphate buffered saline (DPBS, pH 7.4) and 3-(4,5-dimethylthiazol-2-yl)-2,5-diphenyltetrazolium bromide (MTT) were purchased from Sigma-Aldrich (Shanghai, China). The live/dead^®^ viability/cytotoxicity kit for mammalian cells was purchased from Invitrogen (Carlsbad, CA, USA). Dulbecco’s modified Eagle medium (DMEM), foetal bovine serum (FBS), penicillin–streptomycin, and 0.25% (w/v) trypsin were purchased from Procell (Wuhan, China). Bovine serum albumin (BSA), Triton X-100, crystal violet and 4′,6-diamidino-2-phenylindole (DAPI) were purchased from Ding Guo Chang Sheng Biotechnology Co. Ltd. (Beijing, China). Both MCF-7 cells and athymic nude mice (BALB/c-nu) were supplied by Medical Center of Xi’an Jiaotong University (Xi’an, China). All other reagents and solvents were of analytical grade and purchased from Sinopharm Chemical Reagent Co. Ltd. (Xi’an, China). All aqueous solutions were prepared using ultrapure water with a resistance of 18.25 MΩ. Culture plates (24-well plates) and T-25 cm^2^ tissue culture flasks were purchased from Thermo Fisher Scientific (Waltham, MA, USA). The following antibodies were used: rabbit polyclonal Ab against CD34 (EP373Y; Abcam, Cambridge, UK) at a dilution of 1:500, rabbit polyclonal Ab against vascular endothelial growth factor (VEGF)-A (EP1176Y; Abcam) at a dilution of 1:100, rabbit polyclonal Ab against platelet-derived growth factor (PDGF)-B (EPR6834; Abcam) at a dilution of 1:50, rabbit polyclonal Ab against basic fibroblast growth factor (bFGF) (#36769; Signalway Antibody, College Park, MD, USA) at a dilution of 1:50 and a horseradish peroxidase-conjugated goat anti-rabbit antibody (GB23303; Goodbio Technology, Wuhan, China) at a dilution of 1:200. The diaminobenzidine (DAB) Colour Kit was purchased from DAKO (K5007; Copenhagen, DK). Recombinant human endostatin (Endostar) was purchased from Xian Sheng Mai De Jin Co. Ltd. (Shandong, China), and Bevacizumab (Avastin) was purchased from Shanghai Roche Pharmaceuticals Co. Ltd. (Shanghai, China).

### Synthesis of HECS–GMA hydrogels

#### Synthesis of HECS–GMA

Three types of HECS–GMA were synthesized according to a method reported previously with some changes [24]. Chitosan was prepared by adding chitosan (10 g, 62 mmol) slowly to a 50% w/v NaOH (16.7 g) solution and mechanically stirring at room temperature to obtain alkaline chitosan. The alkaline chitosan was stored at 4 °C overnight. Then 133 mL of isopropanol was added and the mixture was stirred mechanically for 90 min at 85 °C under reflux using a Dean–Stark trap. After this step, 18.3 mL of ethylene chlorohydrin, dissolved in isopropanol (33.3 mL), was added to the aforementioned mixture and stirring continued for 5 h under reflux at 65 °C. The desired product of HECS was obtained after filtration, washing with ethyl alcohol, dialysis against water and lyophilisation. The chemical structure of HECS was confirmed from the ^13^C NMR (Bruker, Karlsruhe, Germany) spectrum. Solutions of GMA (0.98, 1.46, and 1.95 mmol) in dimethyl sulphoxide (10 mL) were added to HECS solution (1 g in 90 mL water). The three reaction mixtures were incubated at 70 °C for 6 h with stirring. The product from each mixture was purified by dialysis (molecular weight cut-off: 3500) for 3 days against distilled water. The aqueous solutions were filtered, evaporated, and lyophilized. The three products were confirmed by ^1^H NMR spectra.

#### Synthesis of HECS–GMA hydrogels

Each of the three HECS–GMAs (35 mg), prepared as described above, was dissolved in 1 mL of distilled water. Irgacure 2959 [0.1% (w/w)] was added to the solutions. Polymerization was initiated by ultraviolet irradiation for 120 s at approximately 400 mW/cm^2^. According to the mole percentages of grafted GMA in HECS (20, 30 and 40%, relative to the number of repeating HECS units), the corresponding hydrogels were designated as HECS–GMA20, HECS–GMA30, and HECS–GMA40, respectively.

### Characterization of the HECS–GMA hydrogels

#### Rheological analysis

Both rheological measurements (time sweep test and oscillation frequency sweep test) were performed on a Malvern Kinexus Pro + rotational rheometer (Malvern, UK) by using parallel plate geometry with a diameter of 20 mm and a sample gap of 1 mm. For the time sweep test to monitor the gelation process, 320 µL of fresh solution (ensuring a gap size of 1 mm) was irradiated by an Omnicure S2000 lamp, which was auxiliary equipment for the rheometer, for 120 s at a frequency of 1 Hz and a strain of 1%. For the oscillation frequency sweep test, HECS–GMA hydrogels were exposed to a shear frequency increasing from 0.1 to 10 Hz while maintaining the shear amplitude at 1% and the temperature at 37 °C. As the shear frequency changed, variation of the elastic (storage, G′) and viscous (loss, G′′) moduli was observed.

#### Swelling behaviour

To investigate the swelling ratios, lyophilized HECS–GMA hydrogels were weighed in the dry state (W_d_) and then immersed in Dulbecco’s phosphate-buffered saline (DPBS, pH 7.4) at 37 °C. At regular time intervals, samples were removed to obtain the wet weight (W_s_) after removal of the water on the surface. This step was repeated until the weight did not change. The swelling ratios were inferred from the following formula: swelling ratio = (W_s_ − W_d_)/W_d_.

#### Morphology

Scanning electron microscopy was performed on the HECS–GMA hydrogels (lyophilized to maintain the porous structure without any collapse) to obtain information on the pore structure. The lyophilized hydrogels were sputter-coated with gold and investigated by using a hitachi (Tokyo, Japan) S-3400N scanning electron microscope at an accelerating voltage of 20 kV.

#### 3D and 2D cell cultures

MCF-7 cells were maintained in DMEM supplemented with 10% FBS and 1% penicillin–streptomycin at 37 °C in 5% CO_2_. The disc-like sterile HECS–GMA40 hydrogels with a certain thickness of 2–3 mm and a diameter of 15 mm close to the 24-well culture plate were infiltrated with 0.5 mL of cell suspension (10^6^ cells/mL) and incubated at 37 °C for 4 h. 1 mL of culture medium was added to each disc. The culture medium was replaced every other day. Traditional 2D cell cultures with the same number of cells were used as a control.

### Cell morphology

To evaluate the attachment of MCF-7 cells onto HECS–GMA hydrogels, cells were seeded onto the hydrogels and incubated for 1, 4 and 7 days in 24-well culture plates. The hydrogels (HECS–GMA40) were then washed gently with DPBS to remove non-adherent cells. The hydrogels were viewed with an inverted microscope (Leica, Wetzlar, Germany). The experiments were repeated three times.

Cell morphology was observed with a confocal laser scanning microscope (CLSM, Leica) using fluorescent staining. After 1, 4 and 7 days of cultivation, MCF-7 cells in the HECS–GMA hydrogels were rinsed with DPBS twice, fixed in 4% paraformaldehyde for 20 min and permeabilised with 0.2% Triton X-100 for 10 min. To prevent non-specific labelling, the cells were treated with blocking buffer (1% BSA) for 20 min and then washed with DPBS three times. The actin cytoskeleton was stained with 5 μg/mL FITC-phalloidin for 1 h. Nuclei were stained with DAPI for 10 min and visualized by CLSM. The experiments were repeated three times.

### Cell viability

The viability of cells in 3D and 2D cultures was determined on days 1, 4 and 7 using a live/dead^®^ viability/cytotoxicity kit according to the manufacturer’s recommendations. Calcein AM (0.5 μM) and ethidium homodimer-1 (1 μM) were used to stain viable and dying cells, respectively. The hydrogel/cell constructs were observed using an inverted fluorescence microscope (Leica, Wetzlar, Germany). The experiments were repeated three times.

### Cell proliferation

Cell proliferation was evaluated using the MTT assay. After 1, 4 and 7 days of incubation, cell culture supernatants were removed and the hydrogel/cell constructs were washed completely with DPBS culture medium (0.9 mL). Then, 0.1 mL of MTT (5 mg/mL) was added into each sample well and incubated for 4 h at 37 °C. After removal of the culture medium, the blue formazan crystals were dissolved in 1.0 mL of dimethyl sulphoxide with shaking for 30 min. The absorbance of the solutions was measured at 570 nm using an enspire microplate reader (PerkinElmer, Shelton, CT, USA). The blank control groups lacked cells and were treated identically to the experimental groups. Each experiment was repeated three times.

### Cell migration

The migration abilities of MCF-7 cells were investigated using transwell (8 μm, EMD Millipore, Billerica, MA, USA) assays. After incubation for 1, 4 and 7 days, MCF-7 cells were digested and rinsed with DPBS, and suspended at 5 × 10^6^ cells/mL in DMEM containing 0.2% BSA. Cell suspensions (100 μL) were placed in each upper transwell chamber and 500 μL of medium containing 20% FBS was used as the chemoattractant in the lower chamber. After 24 h, cells failing to invade through the pores were removed using a cotton swab. Invasive cells on the lower surface of the membrane were fixed with methanol, stained with 0.1% crystal violet and counted under an inverted microscope. The experiment was repeated three times.

### Induction of in vivo xenograft tumours

All animal experiments were performed according to the Chinese Ministry of Public Health Guide and US National Institutes of Health guidelines. The tumour-forming capabilities of MCF-7 cells cultured in HECS–GMA40 hydrogels and 2D monolayers were examined following subcutaneous injection of the cells into five BALB/c nude mice. MCF-7 cells were cultured for 7 days in T-25 cm^2^ tissue culture flasks or without HECS–GMA40 hydrogels and harvested. Cells (1 × 10^7^) were resuspended in 100 μL of DMEM and injected into the right (3D) and left (2D) flank of each 4–5-week-old mouse. The mice were kept in a specific pathogen free facility with a 12-h light/dark cycle and had free access to food and water. Tumour volumes were measured with a vernier calliper in two dimensions every 5 days. Tumour volumes were calculated using the formula ab^2^/2, where a and b are the largest and smallest diameters, respectively. After 6 weeks, the animals were sacrificed and the tumours were harvested.

### IHC staining

IHC staining was performed by the standard streptavidin-peroxidase method. The nude mouse xenograft tumours were fixed with 4% paraformaldehyde for 24 h followed by decalcification, dehydration and embedding in paraffin. After hematoxylin and eosin staining for tumour confirmation, IHC staining was performed on 4-µm sections. The slides were incubated with the primary antibodies for CD34 (EP373Y; dilution of 1:500), VEGF-A (EP1176Y; dilution of 1:100), PDGF-B (EPR6834; dilution of 1:50), bFGF (#36769; dilution of 1:50). A horseradish peroxidase-conjugated goat anti-rabbit antibody (GB23303; dilution of 1:200) was used as the secondary antibody overnight at 4 °C. Negative control slices were incubated in the antibody solution but without antibodies. After staining with 3,3-diaminobenzidine, the slices were counterstained with hematoxylin, dehydrated, cleared and mounted. The stained slides were observed and photographs were obtained using an invenio 1D microscope camera (Deltapix, Smorum, Denmark).

To quantify angiogenesis, an anti-CD34 antibody was used to stain microvessel epithelial cells. Positive reactions were indicated by a reddish-brown precipitate in the cytoplasm. The intensity of positive staining was measured through Image-Pro Plus 6.0 software (Media Cybernetics, Rockville, MD, USA). All images were taken using the same microscope and camera sets. The intensity of positive staining in blood vessels or cells was valued by the mean integrated optical density (mean IOD) according to the following formula: mean IOD = IOD/area of the tumour section.

### Effect of drugs on in vivo 3D culture xenograft tumours

All animal experiments were performed according to the Chinese Ministry of Public Health Guide and US National Institutes of Health guidelines. MCF-7 cells were cultured for 7 days in T-25 cm^2^ tissue culture flasks with HECS–GMA40 hydrogels and harvested. Cells (1 × 10^7^) were resuspended in 100 μL of DMEM and injected into the right flank of each 4–5-week-old BALB/c nude mice. The 20 mice were kept in a specific pathogen free facility with a 12-h light/dark cycle and had free access to food and water. When tumours became palpable (100–200 mm^3^), 15 mice were randomized into three groups of five: saline control [1 mL/day, intraperitoneally (i.p.)], Endostar (10 mg/kg/day, i.p.) and Bevacizumab (5 mg/kg/twice weekly, i.p.). Tumour volumes and body weights were measured twice weekly. After 2 weeks, the animals were sacrificed and the tumours were harvested. Tumour volumes were calculated as described above. The rate of tumour inhibition in each treatment was evaluated according to the following formula: tumour inhibition rate (%) = (average tumour volume of control group − average tumour volume of experimental group)/average tumour volume of control group × 100. IHC staining was performed as described above.

### Statistical analysis

Data are presented as the mean ± standard deviation. Student’s *t* test was used to determine the statistical significance between two groups. SPSS 18.0 for Windows (IBM, Chicago, IL, USA) was used for the statistical analysis. Data were considered as statistically significant when P < 0.05.

## Results

### Synthesis of HECS–GMA hydrogels

**Fig. 1:**
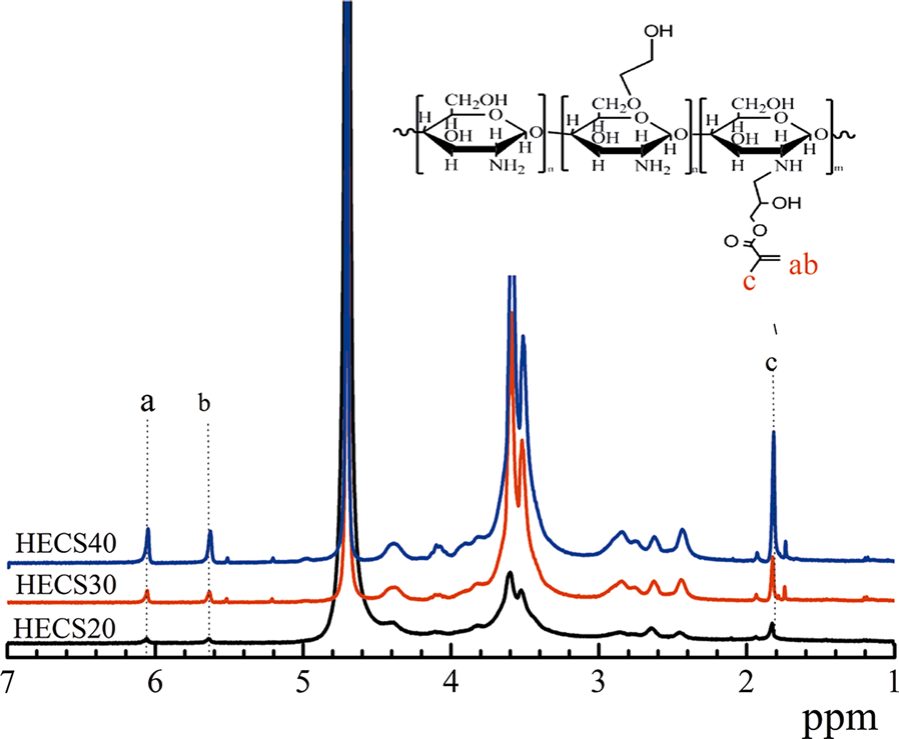
^1^H NMR spectra of three kinds of HECS–GMA hydrogels

**Fig. 2:**
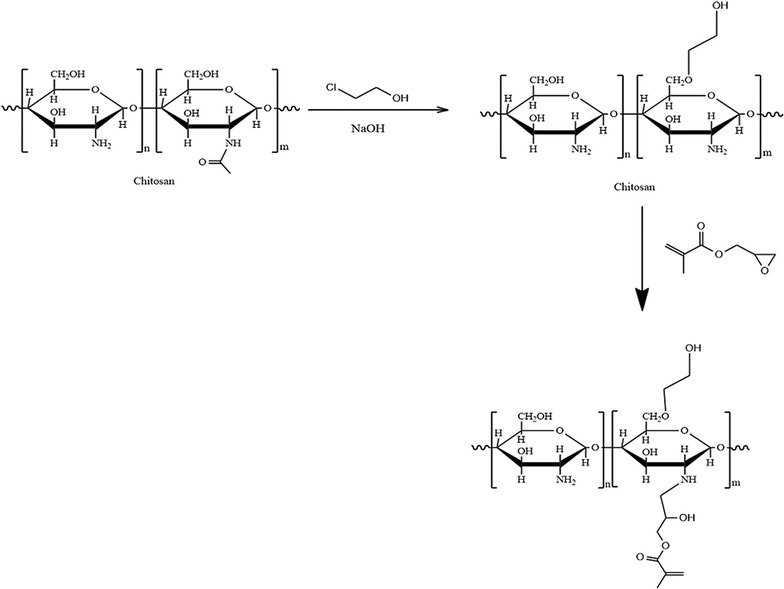
Reaction process of HECS–GMA hydrogel

Chitosan is a cationic polymer that is water-soluble only under acidic conditions. To achieve the aim of dissolving chitosan in water at any pH, HECS was prepared. The hydroxyethyl modification of chitosan reduces its hydrogen bonding and crystallization properties. Consequently, its aqueous solubility was improved. GMA was then conjugated to the amine group of HECS to insert a reaction site for gel formation. The synthesis of HECS was characterized by ^13^C NMR measurements. The synthesis of HECS–GMA was confirmed using the ^1^H NMR spectrum. This spectrum showed new peaks at 1.83 (c), 5.64 (b), and 6.06 (a) ppm that were assigned to GMA. a and b were the chemical shifts of C=C in GMA, and c was the chemical shift of methyl in GMA (Fig. 1). This demonstrated that the HECS–GMA was synthesized successfully. In addition, the substitution degree (14, 20 and 32%, respectively) was calculated by integration. The reaction process is shown in Fig. 2.

### Characterization of HECS–GMA hydrogels

#### Mechanical properties

**Fig. 3:**
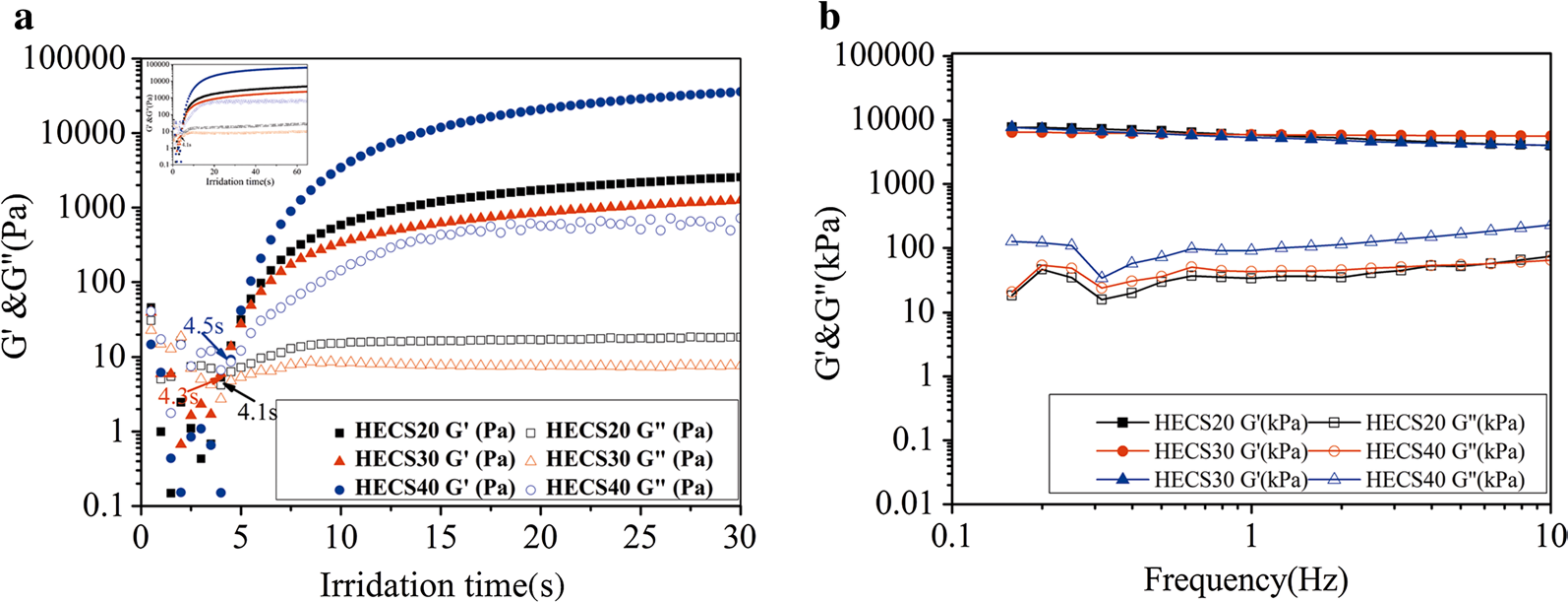
**a** Time sweep test and **b** oscillation frequency sweep

Gel point is an important index to evaluate the speed of hydrogel formation. The time sweep test was utilized to observe the process of gel formation (Fig. 3a). Hydrogels were formed after 7.5 s irradiation by the Omnicure S2000 lamp irrespective of the degree of GMA substitution. The oscillation frequency sweep results demonstrated that HECS–GMA hydrogels had outstanding elastomeric properties because the storage (G′) moduli were much higher than the loss (G′′) moduli over the entire frequency range (Fig. 3b). The G′′/G′ ratios of HECS–GMA20, HECS–GMA30 and HECS–GMA40 were 0.0064, 0.0075 and 0.021, respectively. The G′ moduli were unchanged, whereas the substitution degrees of GMA increased as the G′′ moduli increased. Thus, increasing amounts of GMA conjugated to HECS resulted in a smaller the G′′/G′ ratio. These results indicate that the degree of substitution of GMA plays a predominant role in the rheological properties of hydrogels. The HECS–GMA40 hydrogel may be suitable as a 3D tumour model.

#### Swelling behaviours

**Fig. 4:**
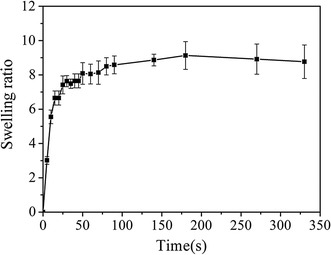
Swelling kinetics of HECS–GMA hydrogels

The results of the swelling experiments are shown in Fig. 4. The curves pointed to a limited, but very rapid uptake of water during the initial 50 s. A slight variation occurred as the incubation time was prolonged. The hydrogels reached their equilibrium swelling ratio of 9.1 ± 0.69 with in 3 min. The three types of HECS–GMA hydrogels showed the same curve. Because of the rapid swelling, the hydrogels attained their equilibrium state during the preliminary swelling process. Therefore, cancer cells can evenly distribute in the porous structure.

#### Hydrogel morphology

**Fig. 5:**
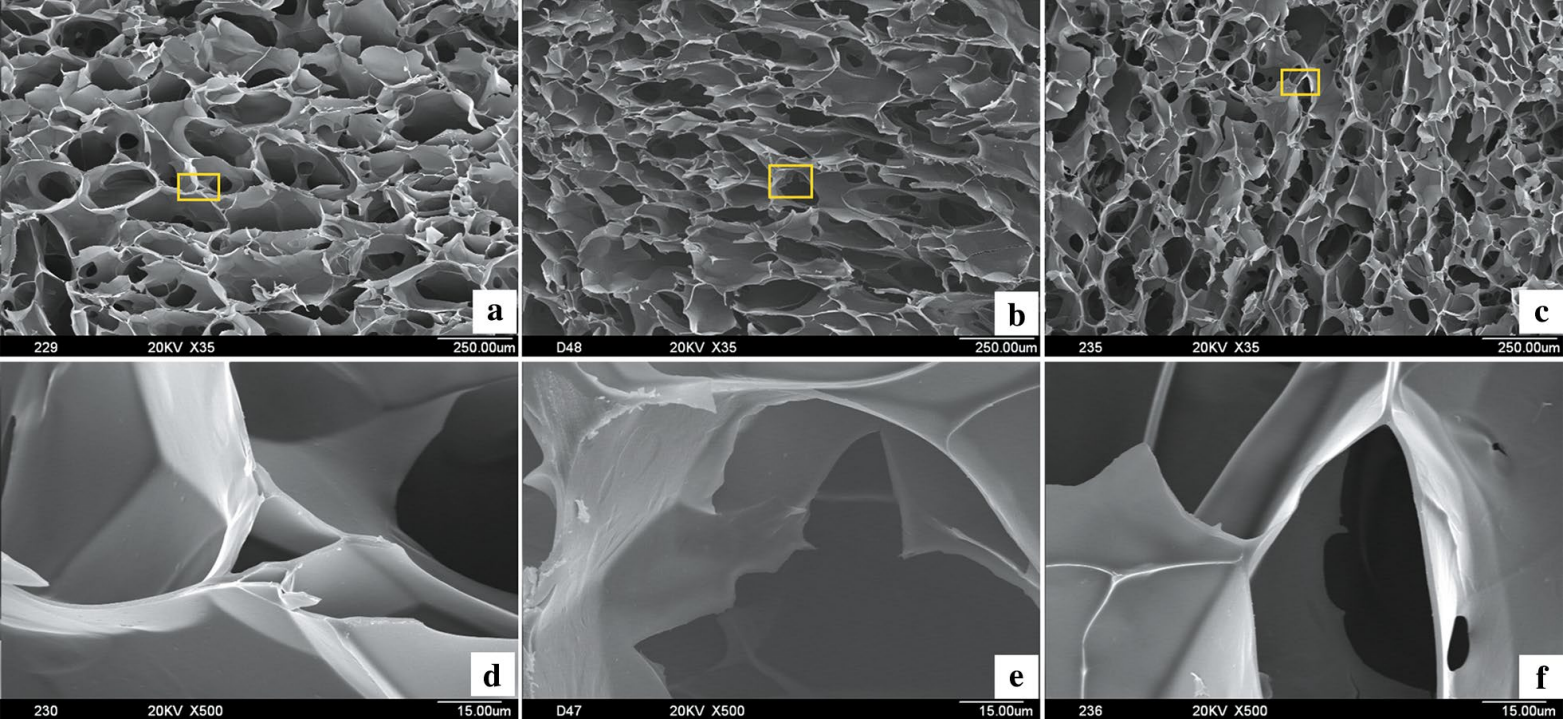
SEM images of three kinds of HECS–GMA hydrogels. **a**–**c** The HECS–GMA20, HECS–GMA30 and HECS–GMA40, respectively (*scale bar* 250 μm). **d**–**f** The corresponding magnified pictures at a *scale bar* of 50 μm

Hydrogel scaffolds possess a particular internal architecture and channels that provide sites for cell adhesion, growth, proliferation, and delivery of nutrition [26]. Scanning electron microscopy images indicated that all hydrogels had uniform porous structures, smooth pore walls, and pore sizes (260, 200 and 180 µm, respectively) that decreased with increasing substitution of GMA (Fig. 5). These properties demonstrate that HECS–GMA hydrogels are an excellent scaffold for the 3D tumour model. A large pore size would cause cells to leak out of the hydrogel. According to the morphologies of the three different hydrogels produced, HECS–GMA40, with a mean pore size of approximately 180 µm, was chosen as the 3D tumour model.

### Cell morphology and viability

**Fig. 6:**
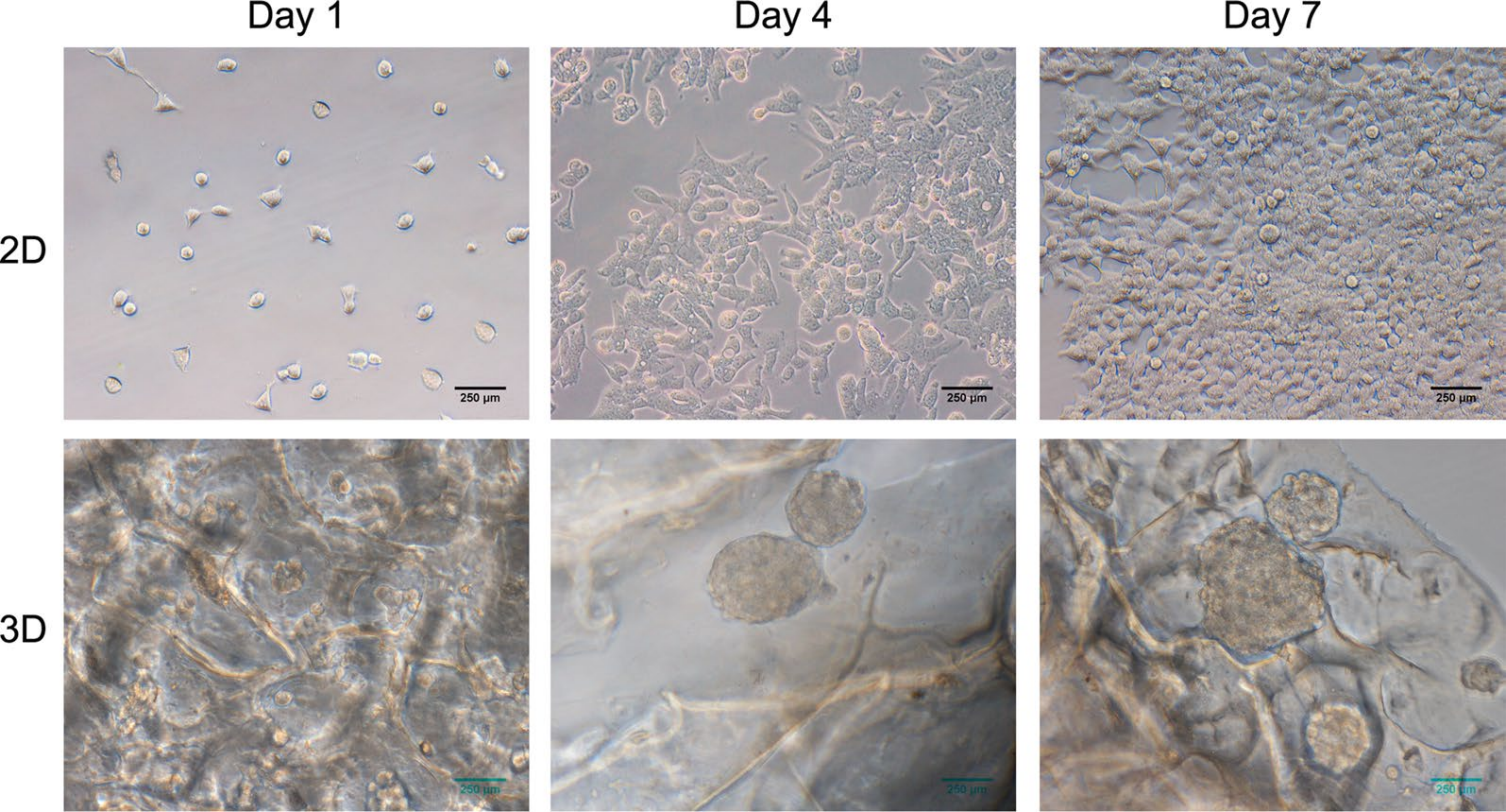
MCF-7 cells in 2D cultures and hydrogels/cells in 3D cultures on day 1, 4 and 7 were viewed in an inverted microscope

**Fig. 7:**
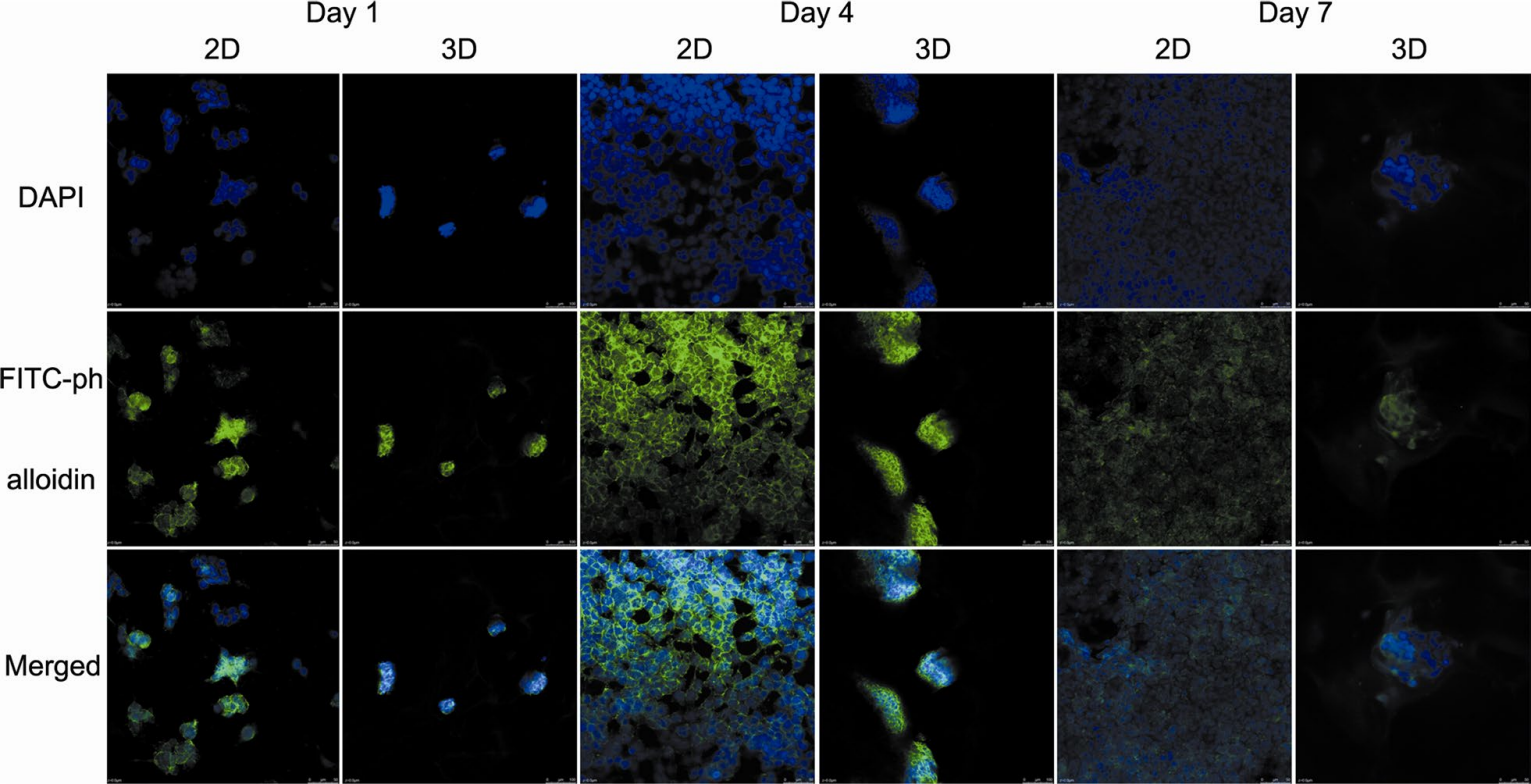
CLSM images of MCF-7 cells in 2D and 3D cultures on day 1, 4 and 7 (*scale bar* 50 μm)

MCF-7 cells in 2D cultures exhibited round, oblong, or irregular morphologies in flat plates, whereas cells in 3D cultures were spherical with their size increasing gradually with time (Fig. 6). Because hydrogels are translucent, some of the cell spheroids could not be seen. To observe the cells in both 2D and 3D cultures more clearly, the actin cytoskeleton was stained green with FITC-phalloidin and the cell nucleus was stained blue with DAPI (Fig. 7). During the scanning process, the diameters of spheroids composed of cells in 3D cultures were constantly varied, which showed that spheroids with 3D structure were implanted in the hydrogels. Figure 7 shows that the MCF-7 cells cultured in 2D proliferated more rapidly than those in 3D hydrogels at 1 and 4 days; however, the proliferation of MCF-7 cells cultured in 2D was slower than that of cells in 3D hydrogels at 7 days. This phenomenon was consistent with the quantitative cell proliferation data.

**Fig. 8:**
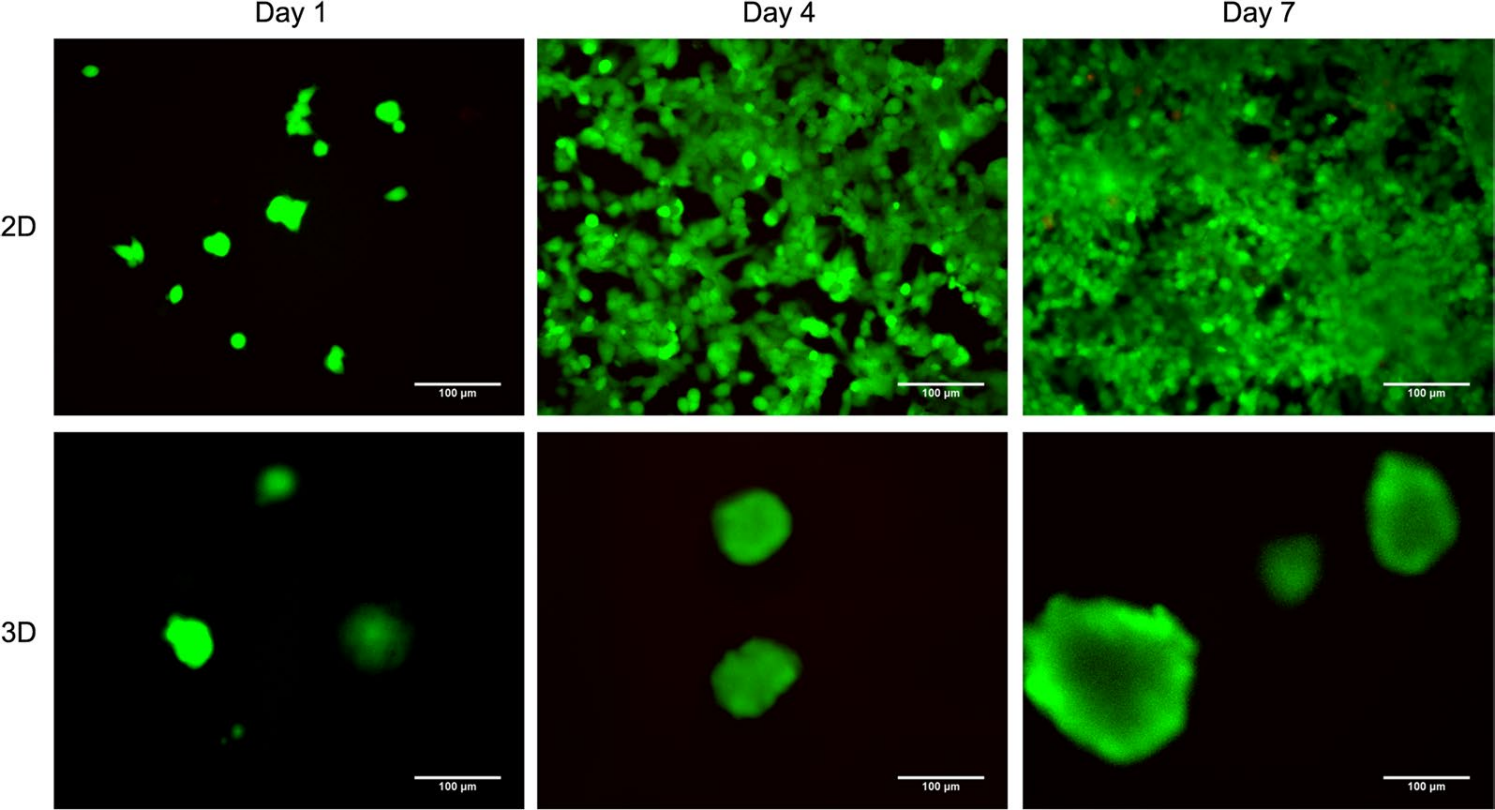
Viability of MCF-7 cells in 3D and 2D cultures

To investigate the cytocompatibility of the hydrogels, the viability of MCF-7 cells grown in 3D and 2D culture conditions was evaluated by a live/dead staining assay. The intrinsic fluorescence of hydrogels interfered with the results. However, over time, red fluorescence (indicative of death) in the 2D cultures and the core of the tumour spheres in the 3D cultures became apparent, especially on the seventh day (Fig. 8). The viability of MCF-7 cells in the body is mainly determined by the transport of nutrients and oxygen. The core of the tumour spheres in the 3D cultures showed lower viability than the periphery because of the lack of nutrients and oxygen, which is consistent with the cell growth pattern in vivo. These results demonstrate that HECS–GMA hydrogels can mimic in vivo microenvironment for culture of cells.

### Cell proliferation and migration

**Fig. 9:**
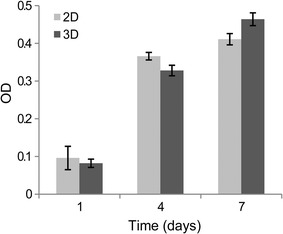
Proliferation kinetics of MCF-7 cells in 2D and 3D cultures

The proliferation of MCF-7 cells in HECS–GMA hydrogels was investigated through the MTT assay. On day 1, the difference between 2D and 3D cultures was not significant (0.096 ± 0.031 vs. 0.082 ± 0.011, respectively) (P > 0.05). However, over time, the discrepancy between 2D and 3D cultures became significant (P < 0.05) (Fig. 9). These results show that cells in the 2D cultures had higher proliferation rates than those in the 3D cultures initially, but the 3D cultures maintained a longer proliferation phase. This finding was consistent with previous studies [27].

**Fig. 10:**
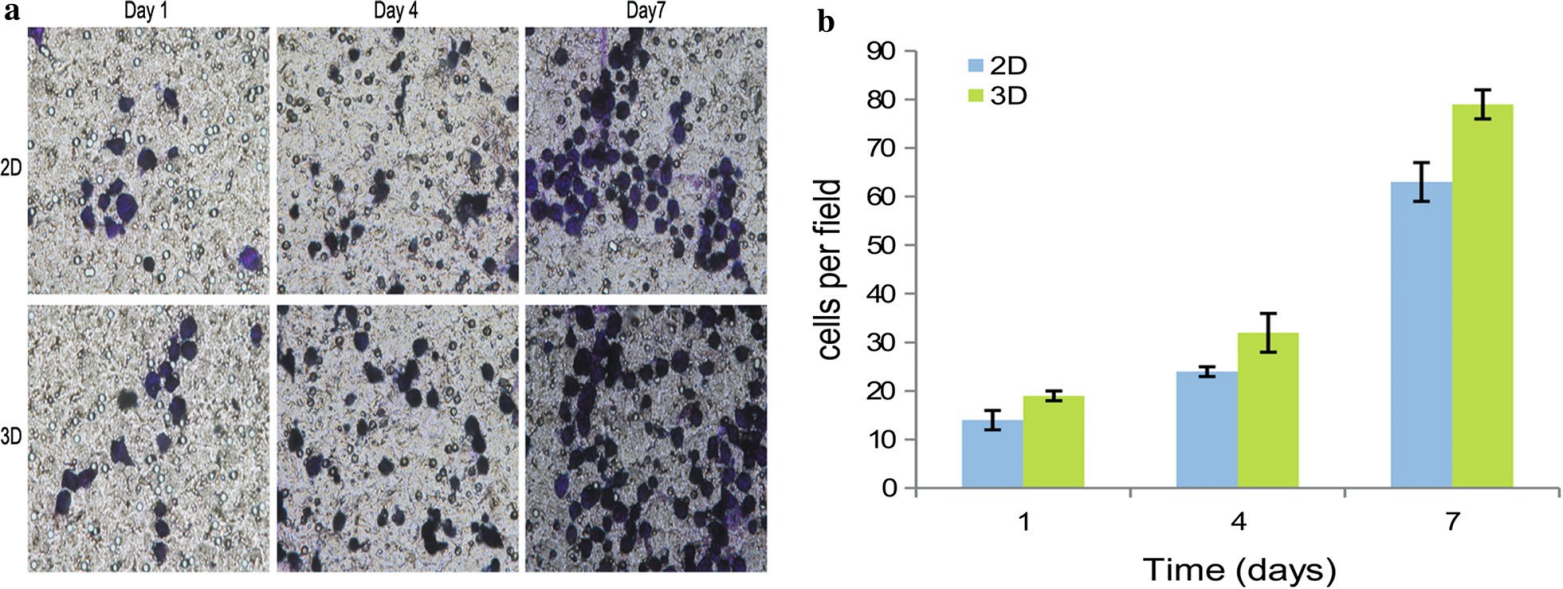
Migration ability of MCF-7 cells in 2D and 3D cultures. **a** Crystal violet staining images of migrated cells and **b** corresponding quantitative results

The migration ability of MCF-7 cells was investigated using a transwell assay. Cell counts were performed for five random high-power fields (Fig. 10a). In 3D cultures, the number of cells was 19 ± 1, 32 ± 4 and 79 ± 3, versus 14 ± 2, 24 ± 1 and 63 ± 4 cells in the 2D cultures at 1, 4 and 7 days, respectively (P < 0.05 at each day) (Fig. 10b). This indicates that the malignant phenotype of MCF-7 cells in the 3D cultures increased more than that in the control group. The increased migration of cells in 3D cultures may be related to the pore size and stiffness of the hydrogels, along with changes in the expression of signalling molecules [28, 29].

### Xenograft growth

**Fig. 11:**
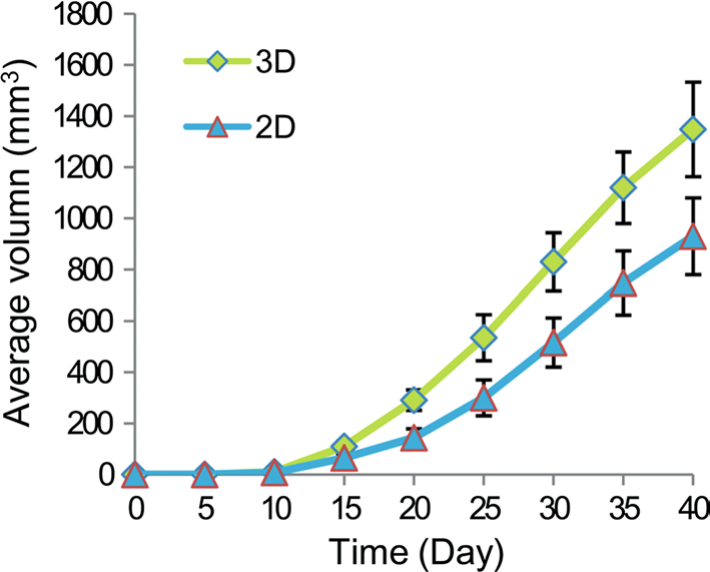
The average tumour volume of the xenografts formed by MCF-7 cells pre-cultured in 2D and 3D systems

To assess the in vivo tumourigenic capabilities of MCF-7 cells grown in both 3D and 2D cultures, cells cultured for 7 days were injected subcutaneously into BALB/c nude mice. By 10–15 days after injection, subcutaneous tumours could be observed and palpated. The tumours formed from cells pre-cultured in 3D hydrogels grew far more rapidly than those from cells pre-cultured in 2D monolayers throughout the whole growth process. The tumour volumes on day 15 after injections were 110 ± 20 and 65 ± 15 mm^3^, respectively. The tumour volumes increased almost linearly with time from day 15 to day 35 after injections regardless of pre-culture conditions. On day 30 after injections, the average volume of the tumours formed from 3D-and 2D-cultured cells was 830 ± 114 and 516 ± 96 mm^3^, respectively. After 6 weeks, the tumours were large enough to harvest. The average tumour volume of tumours derived from 3D cultures was 1348 ± 185 mm^3^, which was larger than the 930 ± 150 mm^3^ for cells from 2D cultures (P < 0.05) (Fig. 11).

CD34 is a high-specificity marker of vascular endothelial cells that can directly reflect the micro vascular density. VEGF-A has activity for angiogenesis or blood vessel formation, primarily through its interactions with the VEGFR1 and -R2 receptors found on the endothelial cell membrane. PDGF-B is known as a mitogen and chemotactic agent for fibroblasts and smooth muscle cells, and an inducer of extracellular matrix protein synthesis. bFGF stimulates the activity of fibroblasts, endothelial cells, smooth muscle cells, and neurons, and induces angiogenesis via stimulation of VEGF expression. These mechanisms are all involved in tumour angiogenesis.

**Fig. 12:**
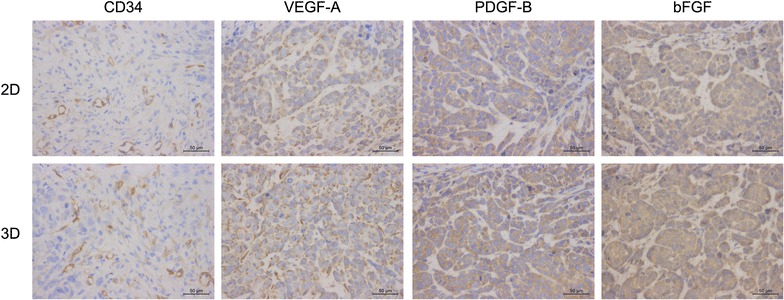
Microscopically, the expression of CD34, VEGF-A, PDGF-B and bFGF of the xenografts formed by MCF-7 cells pre-cultured in 2D and 3D systems. *Reddish*-*brown precipitate* indicates positive staining. Original magnification ×400

IHC staining showed that CD34 was expressed mainly in the epithelial cells of microvessels, whereas VEGF-A, PDGF-B and bFGF were expressed mainly in the cytoplasm of tumour cells (Fig. 12). Semi-quantitative mean IOD analysis indicated more positive staining in the xenografts derived from the 3D culture than in those derived from the 2D culture. The differences in CD34 and VEGF-A between the two groups were statistically significant (P < 0.05), but the differences in PDGF-B and bFGF were not (P > 0.05) (Table 1).

**Table 1:** Average optic density in xenografts formed by MCF-7 cells pre-cultured in 2D and 3D systems (IOD/area)

Group	n	CD34	VEGF-A	PDGF-B	bFGF
2D	5	0.087 ± 0.001	0.131 ± 0.003	0.156 ± 0.005	0.165 ± 0.007
3D	5	0.104 ± 0.003^a^	0.148 ± 0.002^a^	0.159 ± 0.006^b^	0.167 ± 0.004^b^

Data presented as mean ± SD

^a^P < 0.05

^b^P > 0.05

### Antitumour activity of Endostar or Bevacizumab in the 3D culture xenograft model

The mean tumour volume of the control group was 630 ± 94 mm^3^, which was significantly higher than that following treatment with Endostar (320 ± 88 mm^3^) (P < 0.05) but not different from that after treatment with Bevacizumab (448 ± 93 mm^3^) (P > 0.05), respectively. The degree of tumour inhibition was 49.2% and 28.9% in the Endostar and Bevacizumab groups at the end of treatment, respectively.

**Fig. 13:**
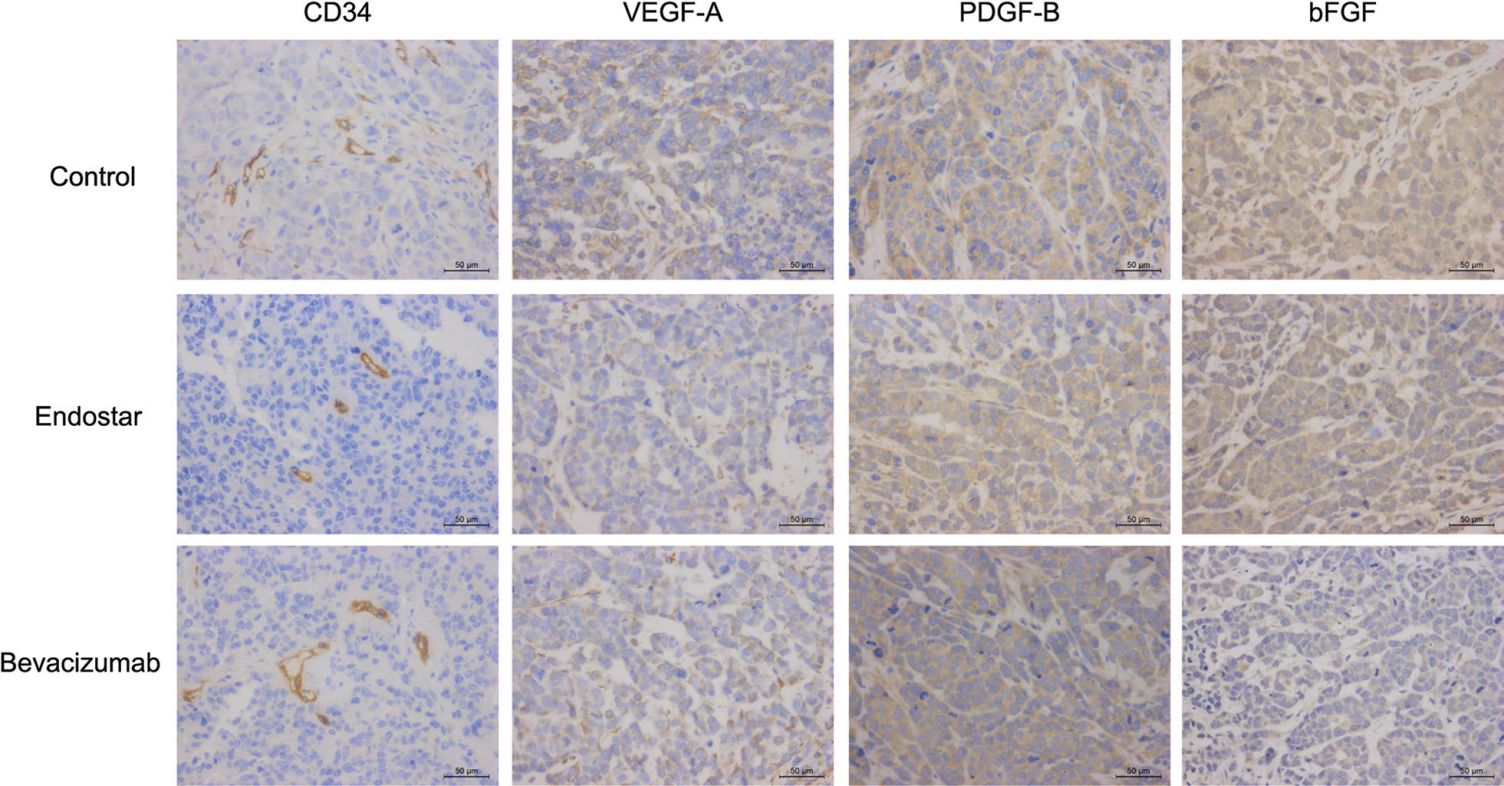
Microscopically, the expression of CD34, VEGF-A, PDGF-B and bFGF of the xenografts formed by MCF-7 cells pre-cultured in 3D systems were affected by Endostar and Bevacizumab treatments (×400)

The expression of CD34, VEGF-A, PDGF-B, and bFGF is shown in Fig. 13. CD34 expression in the Endostar and Bevacizumab groups was lower than that in the control group (0.070 ± 0.003 and 0.074 ± 0.004 vs. 0.082 ± 0.002, respectively; P < 0.05). The expression of VEGF-A in the Endostar and Bevacizumab groups was 0.116 ± 0.003 and 0.119 ± 0.005, respectively, which was significantly lower than that in the control group (0.134 ± 0.006, P < 0.05). bFGF expression was increased in the Bevacizumab group compared to the control group (P < 0.05), whereas statistical significance was not found between the Endostar and control groups (P > 0.05). PDGF-B expression was similar in all groups (P > 0.05) (Table 2).

**Table 2:** Effects of Endostar and Bevacizumab on xenografts formed by MCF-7 cells pre-cultured in 3D systems (IOD/area)

Group	n	CD34	VEGF-A	PDGF-B	bFGF
Control	5	0.082 ± 0.002	0.134 ± 0.006	0.128 ± 0.008	0.157 ± 0.008
Endostar	5	0.070 ± 0.003^a^	0.116 ± 0.003^a^	0.124 ± 0.009^b^	0.155 ± 0.010^b^
Bevacizumab	5	0.074 ± 0.004^a^	0.119 ± 0.005^a^	0.126 ± 0.011^b^	0.140 ± 0.007^a^

^a^P < 0.05

^b^P > 0.05

## Discussion

Breast cancer is a heterogeneous disease with complex tissue environments that affect cancer initiation, metastasis, angiogenesis, and resistance to therapy [30, 31]. The discovery of tumour angiogenesis opened a new path in fighting cancer that increased the effectiveness of standard chemotherapy, or even replaced it, by offering better patient outcomes [32]. Over the past decade, extensive studies of 3D cultures have demonstrated differences compared to the behaviour of cells on 2D surfaces [33]. A good tumour microenvironment model that closely simulates the real tumour construct would not only enhance the study of physiological mechanisms in vitro, but also dramatically improve the translation of novel chemotherapeutics from in vitro to in vivo testing [13].

Hydrogels are cross-linked networks of the same or different types of polymers with a high capacity for water absorption [34]. Natural gels derived from extracellular matrix components and other biological sources are biocompatible and inherit the bioactivity of their starting material, which is something that synthetic polymers usually lack [35–37]. To model the breast cancer microenvironment, and study the growth of breast cancer MCF-7 cells and the targeting of anti-angiogenic agents in 3D cultures, we constructed 3D HECS–GMA hydrogels using chitosan as a platform. The modification of chitosan to HECS–GMA hydrogels increased the water solubility and viscoelasticity, while enabling control of the pore size and enhancing biocompatibility, which was different from other methods [38]. Additional research is needed to explore the ability of cross-linking to prevent dissolution of the hydrogel polymer chains by rheological processes [39]. The G′ and G′′ values of our hydrogels had a low frequency dependency, indicating that the hydrogel networks were highly stable. These results indicate that the proportion of energy stored elastically in the hydrogels was much higher than that of dissipated energy. Furthermore, the swelling ratio of the hydrogels was small, indicating that the network had a significant influence on the swelling ratio.

The pore sizes of the hydrogels were suitable for material exchange by cells by providing the necessary space and nutrition channels. The HECS–GMA hydrogels achieved unique structural (fibro-porous, non-toxic) and mechanical (high tensile strength with flexibility) properties, providing oxygen and water permeability to support cell adhesion and growth.

In 3D cultures, breast cancer cells formed spheres embedded in the hydrogels. These spheres could be observed with an inverted microscope and by CLSM. The encapsulation of living cells, and chemical cross-linking, provide hydrogels with excellent mechanical strength, which is different from other scaffolds [25, 38]. In 2D cultures, MCF7 cells can initially obtain sufficient nutrients and oxygen to grow quickly. However, there are limitations on proliferation in 2D cultures as the number of cells increases, and space and nutrients are constrained. With increasing time in 3D cultures, cell spheroids grew, and hypoxic cells in the core grew more slowly than peripheral cells. This phenomenon, consistent with tumour growth in vivo, showed that the HECS–GMA hydrogels had good cytocompatibility [40, 41].

To further study the characteristics of 3D cultured cells, their growth in vivo as xenograft tumours and their expression of CD34, VEGF-A, PDGF-B and bFGF were examined. CD34 and VEGF-A expression but not PDGF-B and bFGF expression were significantly different between 3D-cultured cells and 2D cultures. Possible reasons for the lack of difference in the expression of PDGF-B and bFGF include high expression masking the differences, the use of a high antibody concentration, or the short time in 3D culture.

Differential intracellular signalling and transcription of genes has been reported between 2D and 3D cultures [25]. Although 3D cultures are widely used to study the effect of drugs in breast cancer, their use with anti-angiogenic agents is not common [42]. Endostar inhibited breast cancer tumour growth in our study, which was consistent with previous studies [43]. The risks associated with Bevacizumab combination therapy, the lack of a survival benefit, and inconsistencies in the magnitude of the progression free survival time across studies led the US Food and Drug Administration to remove the metastatic breast cancer indication for this drug in November 2011 [44]. Despite this setback, some studies continue to explore a role for Bevacizumab in treating breast cancer [45]. The expression differences that we observed among the various angiogenesis-related growth factors indicate the presence of specific drug targets. However, consistent with the results in clinical practice, our results showed that Bevacizumab was ineffective against breast cancer cells.

Our HECS–GMA hydrogels have several advantages over other 3D scaffolds. They are biocompatible, biodegradable, non-immunogenic and non-inflammatory. These characteristics make it possible to provide an in vitro biomimetic microenvironment for breast cancer culture and drug studies. However, there are some limitations to the hydrogel modelling system. Specifically, the hydrogels will deform and degrade after 7 days, and intrinsic opaqueness and autofluorescence may impair morphologic observation. If the stability of HECS–GMA hydrogels can be elevated, it may become possible to replace xenograft models.

Three-dimensional cultures may promote the expression of CD34, VEGF-A, PDGF-B, and bFGF better than 2D cultures in xenograft tumours in vivo. However, 3D cultures provide an integrated environment, and their effects on cells should be further studied. The gradually increasing use of anti-angiogenic agents makes it important to perform additional research on their efficacy [46]. The 3D culture platform may provide a system suitable for such research, although there may be others factors affecting the efficacy of Endostar and Bevacizumab. Thus, further studies are needed.

## Conclusions

It is increasingly evident that 3D cell culture models are more suitable than the traditional 2D monolayer cultures due to improved cell–cell interactions, cell–extracellular matrix interactions, and cell populations and structures that better resemble the in vivo architecture. In this study, we report that HECS–GMA hydrogels are useful for studying the morphology, viability, proliferation, and migration of breast cancer MCF-7 cells. Breast cancer cells cultured in HECS–GMA hydrogels showed a growth status that mimicked the in vivo situation better than 2D monolayer cultures. The anti-angiogenic efficacy of Endostar and Bevacizumab can be more comprehensively studied and assessed in 3D cultures than in 2D cultures through enhanced expression of angiogenesis-related growth factors in xenograft tumours in vivo. Overall, this study utilized HECS–GMA hydrogels as a 3D culture scaffold to study breast cancer MCF-7 cells. The results indicate that this 3D culture scaffold provides an improved research platform to assess the efficacy of anti-angiogenic agents.

## Abbreviations

2D: two-dimensional
3D: three-dimensional
bFGF: basic fibroblast growth factor
BSA: Bovine serum albumin
CLSM: confocal laser scanning microscopy
DMEM: Dulbecco’s modified Eagle medium
DPBS: Dulbecco’s phosphate-buffered saline
DAPI: 4′,6-diamidino-2-phenylindole
FBS: foetal bovine serum
HECS–GMS: hydroxyethyl chitosan/glycidyl methacrylate
IHC: immunohistochemistry
IOD: integrated optical density
i.p.: intraperitoneally
MTT: 3-(4,5-dimethylthiazol-2-yl)-2,5-diphenyltetrazolium bromide
PDGF: platelet-derived growth factor
VEGF: vascular endothelial growth factor
